# Development of Maize Planting Method Based on Site-Specific Soil Moisture for Improving Seedling Traits in the Northern China Dryland

**DOI:** 10.3390/plants14243859

**Published:** 2025-12-18

**Authors:** Haoming Li, Jialu Sun, Li Yang, Dongxing Zhang, Tao Cui, Kailiang Zhang, Xiantao He, Xinpeng Wang, Yingxuan Wu

**Affiliations:** 1 College of Engineering, China Agricultural University, Beijing 100083, China; 2 Key Laboratory of Soil-Machine-Plant System Technology of Ministry of Agriculture, Beijing 100083, China

**Keywords:** precision planting, dryland, maize, site-specific planting, soil moisture, sowing depth, seedling uniformity

## Abstract

Dryland, which mainly retains rain-fed agriculture, is the main type of farmland in China and widely distributed in the northern regions. Rainfall scarcity limits the development of maize at the seedling stage, which adversely affects the increase in maize yields in this region. A planting method that allows variable sowing depths based on the uneven distribution of soil moisture was proposed in this study. This site-specific planting method which fully utilizes available soil water is able to overcome the above problem. The framework of variable depth seeding suitable for this region was constructed: Within the depth range of 5.5 to 8.5 cm in the soil, maize seeds should be sown to a position with a relative soil moisture of 70%. For some drylands without such moisture conditions, seeds can be placed at the position with the highest relative soil moisture in this depth range. Taking the conventional planting method as the control group, the performance of the variable depth planting method in improving maize seedling growth was evaluated. The results showed that the proposed planting method not only increased the emergence rate and the seedling uniformity by 9.31% and 25.29%, respectively, but also raised the mean leaf number and the mean plant height in the same growth period, having a remarkable effect in improving the maize seedling traits. This planting method is easy to be embedded into precision control systems of the maize planter, and will promote the application of soil moisture-based planting technology and thus increase the yield per hectare of maize.

## 1. Introduction

China covers a large region of dryland farming mainly distributed in the north. It accounts for approximately 56% of the nation’s total land area [1]. Mono-cropping systems consisting mainly of maize [2] are characteristic of this region. Improving maize yields in the dryland of northern China is of great significance, as it contributes to increasing the total national agricultural production and ensuring national food security [3]. Maize typically grows from early April to late September, whereas 60–70% of annual precipitation falls as rain during the summer, from July to September [4]. The mismatch between the rain season and maize growth cycle means that the crop has few opportunities to encounter rainfall during the early seedling growth stages. Since rainwater is the sole water resource in the dryland farming, it causes the water stress to become a primary factor limiting maize seedling growth there [5]. The number of seeds emerging and the uniformity of seedling height are easily reduced by water stress, which ultimately lowers maize yields. Thus, optimizing the development of maize seedlings is overwhelmingly demanded in this region.

Improving maize seedling growth by increasing water use efficiency (WUE) is a reasonable measure under the environment with limited water resources and planting area. According to this, Chinese scientists have proposed various innovative practices for the northern dryland. Some soil surface management techniques, such as conservation tillage and mulching, had been proposed. These techniques help cultivate strong seedlings by reducing soil erosion and water evaporation [6,7,8]. Furthermore, they facilitate the formation of robust and deep root systems, allowing more soil moisture to be utilized by the crop seedlings. Consequently, the growth traits of the seedlings are improved [9]. These research results have made a positive effect on promoting the development of techniques for improving seedling growth by fully utilizing the available soil water. However, there are already large areas of the dryland under conservation tillage in China, but the improvement in seedling growth traits is still insufficient [10]. Work by Kader [11] showed that the major problems brought by mulching are that specialized equipment for installing plastic mulches tends to be costly and natural mulches are not good for weed control. Influenced by the farmland type and unbalanced fertilization, nutrient management techniques also have limited efficiency in improving crop development at the seedling stage [12]. Therefore, to optimize maize seedling growth, continued strategies are still needed to be studied and proposed for the northern dryland of China.

Normally, in northern China, maize planters do not adjust the sowing depth of maize seeds. The seeds are placed at almost the same depth in the soil at any location across a field. This type of maize seeding is the traditional constant-depth planting (TCP) method. But soil water presenting the heterogeneity distribution in farmland is a more prominent feature in dryland compared with other agricultural cropland types [13]. The moisture content of the soil varies horizontally within meters to kilometers and vertically within 10 cm of the surface layer of the soil [14,15], which means that the sowing depth that allows the seeds to obtain the sufficient content of water is variable at different seeding positions. Therefore, in theory, adjusting the sowing depth of maize seeds to place them in a position with adequate water at all times during planting is able to make full use of stored soil water in the dryland of northern China. This variable depth planting (VDP) method, which increases the WUE, has a greater potential to improve maize seedling growth. However, in China, there is still no research to investigate the agronomic value of the VDP method.

The primary objectives of this study were to develop a suitable VDP method for the drylands of northern China, and to validate its superiority in promoting maize seedling growth under conditions of uneven water distribution. This work aims to establish a more rational maize planting strategy to mitigate the limiting effects of scarce rainfall on seedling development in this region.

## 2. Results

### 2.1. Daily Seed Emergence Under Different Relative Soil Moisture and Sowing Depth

The variation in relative soil moisture (RSM) at five sowing depth (SD) levels during the experiment are shown in Table 1. The water stored in the soil evaporated at a decreasing rate with the SD, which suggested that the soil was gradually less disturbed by the external environment. Further, at the end of the experiment, the RSM around sown seeds at 2.5 cm dropped to about 20%, nearly half of the counterpart at 8.5 cm. The longer time of water deficiency made the environment at the shallow SD level unfavorable for maize to carry out various developmental activities.

**Figure 1:**
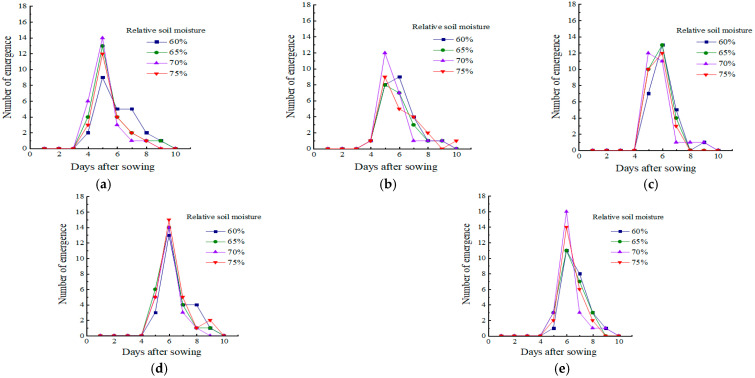
Daily seed emergence at different sowing depth (SD) levels: (**a**) SD 2.5 cm; (**b**) SD 4.0 cm; (**c**) SD 5.5 cm; (**d**) SD 7.0 cm; and (**e**) SD 8.5 cm.

The number of daily seed emergence at four RSM levels is shown in Figure 1. The RSM level of 60% showed a sporadic emergence of seeds after sowing, as the daily variation in the number of seeds emerging under this condition was flatter compared to the other RSM levels. As the RSM increased, maize seeds tended to emerge in a concentrated pattern within a certain day, which may provide more benefits for maize development, as concentrated seedling emergence can ensure simultaneous seed development and reduce vicious competitions for nutrients between adjacent seeds. When RSM reached 70%, most of the seeds sown at all SD levels were able to emerge simultaneously within the fifth or sixth day after sowing. However, the situation was changed when the RSM level reached 75% and the increase in RSM began to negatively affect the concentration of seed emergence.

### 2.2. Effect of Soil Moisture and Sowing Depth on Maize Seedlings

According to the experimental design scheme and results, a binary quadratic polynomial regression model of emergence rate (*ER*, *Y*_1_), mean leaf number (*MLN*, *Y*_2_), mean plant height (*MPH*, *Y*_3_), and plant height uniformity ( $U_{n}$, *Y*_4_) versus relative soil moisture (RSM, *X*_1_) and sowing depth (SD, *X*_2_) was established. The regression equations are shown in Table 2, and the significance analysis results of them are shown in Table 3.

As can be seen from Table 3, the *p*-values for the models on *ER *(*Y*_1_), *MLN (Y*_2_*)*, *MPH *(*Y*_3_), and $U_{n}$ (*Y*_4_) were all less than 0.01, indicating that the fitting degree of the four constructed regression equations was highly significant.

As for the *ER*(*Y*_1_), the *p*-values of *X*_1_, *X*_2_, *X*_1_^2^, *X*_2_^2^, as well as *X*_1_*X*_2_ were less than 0.01, which meant that each regression term had a highly significant effect on the *ER*(*Y*_1_). For *MLN* (*Y*_2_), the *p*-values of *X*_2_, *X*_1_*X*_2_, and *X*_2_^2^ were below 0.01, which indicated that the above-mentioned regression terms had a highly significant effect on *Y*_2_. Moreover, the *p*-value of *X*_1_^2^ was lower than 0.05, suggesting a significant effect of *X*_1_^2^ on *Y*_2_. Whereas, the *p*-value of the *X*_1_ was greater than 0.05, i.e., the *X*_1_ had no significant effect on *Y*_2_. Moving to *MPH* (*Y*_3_), the *p*-value of *X*_1_ was less than 0.01, showing that it had a highly significant effect on *Y*_3_. In addition, the *p*-values of *X*_2_ and *X*_1_^2^ were lower than 0.05, which indicated that these two terms had a significant effect on *Y*_3_. Apart from these, the *p*-values of other regression terms were all >0.05, i.e., they had no significant effect on *Y*_3_. As for $U_{n}$ (*Y*_4_), the *p*-value of *X*_1_ was <0.01, demonstrating that it had a highly significant effect on *Y*_4_. Furthermore, the *p*-values of *X*_2_ and *X*_1_^2^ were <0.05, i.e., the two regression terms had a significant effect on *Y*_4_. However, the other regression terms all had *p*-values above 0.05, which meant that they had no significant effect on *Y*_4_.

To sum up, SD (*X*_2_) was a factor that exerted a highly significant effect on *ER*(*Y*_1_) and *MLN*(*Y*_2_), while RSM (*X*_1_) had a highly significant effect on *ER*(*Y*_1_), *MPH*(*Y*_3_), and $U_{n}$(*Y*_4_). It is true that sowing depth is an important factor affecting maize growth; however, in drylands, RSM apparently acts as a more critical factor, as it had a highly significant impact on more maize growth indexes. The result that the interaction of RSM (*X*_1_) and SD (*X*_2_) also had a highly significant effect on *ER*(*Y*_1_) and *MLN*(*Y*_2_) further suggests that in the process of seeds sown, guaranteeing that the water appropriately supplied to seeds are able to bring a dramatic improvement in seedling growth status.

### 2.3. Significance of Variable Sowing Depth in the Dryland

**Figure 2:**
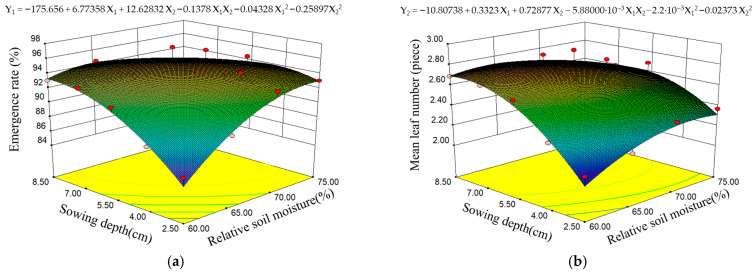
Response surface diagrams of interaction for seedling growth indexes: Effect of the interaction of relative soil moisture (RSM) and sowing depth (SD) on (**a**) emergence rate (ER) and (**b**) mean leaf number (MLN). Abbreviations: ER/Y1, emergence rate; MLN/Y2, mean leaf number; X1, relative soil moisture; X2, sowing depth.

**Figure 3:**
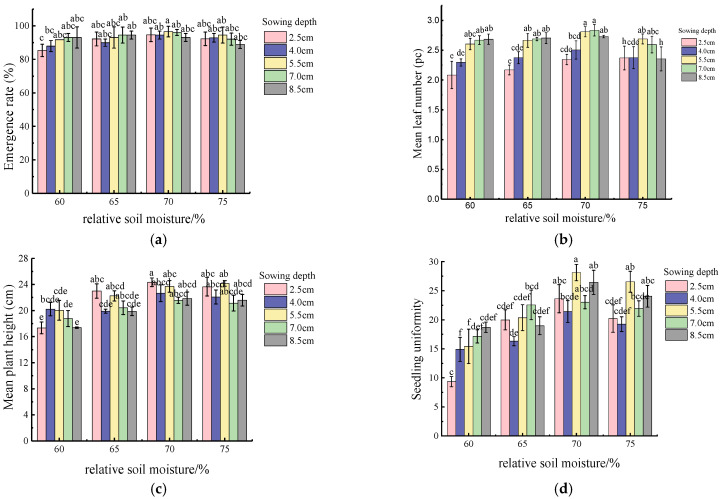
Calculation results of seedling growth indexes at different combinations of relative soil moisture (RSM) and sowing depth (SD): Calculation results of (**a**) emergence rate (ER), (**b**) mean leaf number (MLN), (**c**) mean plant height (MPH), and (**d**) uniformity ( $U_{n}$), respectively. Columns sharing the same letter are not significantly different (*p* ≥ 0.05).

The response surface diagram was obtained after data processing, as shown in Figure 2. In addition, the calculation results of seedling indexes under different levels of sowing depth (SD) and relative soil moisture (RSM) are shown in Figure 3. It is clear that when the value of RSM ranged from 60% to 65%, the increase in SD can significantly improve the number of emerged maize seeds. However, when the RSM was between 70% and 75%, the deeper SD levels showed lower rates of maize emergence (Figure 2a and Figure 3a). If the soil moisture content was deficient, with an increase in SD, the magnitude of the rise in mean leaf number (*MLN*) was quite dramatic. However, when the RSM ranged from 70% to 75%, maize seeds with SD of 5.5 and 7.0 cm presented a more pronounced developmental advantage in terms of leaf number (Figure 2b and Figure 3b). Based on Figure 3c, we can find that when RSM was 60%, the seeds sown at depths in the range of 4.0 to 5.5 cm had better vertical growth. But as the soil moisture increased, the seedlings with sowing depths of 2.5 and 5.5 cm achieved greater mean height than the remaining levels. Figure 3d regarding the calculation results of uniformity ( $U_{n}$) illustrate that when the soil was dry, increasing the SD could improve the $U_{n}$ of maize seedlings in an extremely effective method. While, when RSM was between 70% and 75%, too wet soils made it more difficult for seeds to emerge from deep sowing locations. This resulted in that the $U_{n}$ of maize was no longer optimal at the deeper SD levels.

According to the above analysis, it can be seen that the SD that enables the best result of emergence rate (*ER*), *MLN*, mean plant height (*MPH*), and *U_n_* varies under the soil with different moisture content. It indicates that SD should be adjusted for drylands with heterogeneous water distribution so as to promote the growth of maize seedlings.

### 2.4. Establishment of the Framework of the Variable Depth Planting Method

Both sowing depth (SD) and relative soil moisture (RSM) have important effects on the growth of maize at the seedling stage [16]. However, in farmland, the soil moisture content is difficult to be regulated in real time during actual operations [17], which results that finding the position with an optimal RSM value within the suitable SD range becomes an easier strategy to implement the variable depth planting (VDP) method [5]. Moreover, defining the SD range is able to narrow the detection interval of the soil moisture sensor, which is helpful to improve the work efficiency of the precision planter [18]. Therefore, solving for a suitable SD range at first and finding the optimal RSM value within this range is the main task to construct the framework of the VDP method.

#### 2.4.1. Suitable Range of Sowing Depth

The mean comparison results of maize seedling growth indexes under different SD levels were calculated based on the Duncan test, a post-ANOVA procedure that assigns letter groups to identify significant differences [19]. The results were shown in Table 4. At shallow sowing depths (2.5 cm and 4.0 cm), emergence rate (*ER*), mean leaf number (*MLN*), and uniformity (*U_n_*) that had significant effects on maize yields were obviously lower than the data at deeper sowing locations. In addition, although a lower soil water content corresponds to a deeper optimal sowing depth, Liben’s study [20] showed that the SD of maize in drylands should not exceed 10 cm. Thus, under various soil moisture conditions, the SD range for maize sown based on the VDP method was determined to be between 5.5 and 8.5 cm.

#### 2.4.2. Optimal Value of Relative Soil Moisture

Within the range of suitable sowing depths, the mean comparison results of maize seedling growth indexes under different RSM levels are calculated based on the Duncan test and shown in Table 5. When the overall RSM was between 60% and 65%, maize was in an unfavorable state of development in terms of seedling height and population height uniformity, indicating that this RSM range was not suitable for seed growth. Moreover, if the soil was comparatively wet (RSM of 75%), the difficulty of seedling emergence and nutrient consumption during the period was enhanced, leading to a reduction in *ER* and *MLN*. Thus, the optimal RSM within the SD of 5.5 cm to 8.5 cm was determined to be 70%.

Obviously, if there was a site with an RSM of 70% within the SD of 5.5 cm to 8.5 cm, it would be a comfortable position that allowed the maize seed to reach optimal developmental status during the short seedling stage.

#### 2.4.3. The Framework of the Variable Depth Planting Method

**Figure 4:**
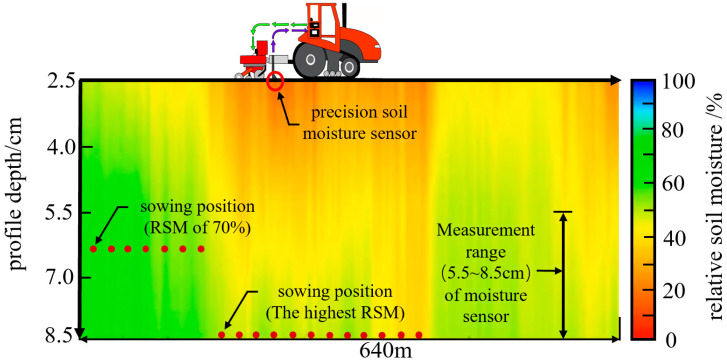
The seeding process with variable depth planting (VDP) method in the dryland. Abbreviations: RSM, relative soil moisture.

The framework of the VDP method for guiding maize mechanized sowing in the dryland of northern China was able to be proposed based on the above analysis [21]. The exact operation process is shown in Figure 4. During the forward movement of a maize planter, a precision sensor mounted in front of the disk openers ensures that RSM information in the depth range of 5.5 to 8.5 cm can be dynamically detected. Maize seeds were sown to positions with 70% RSM based on feedback results of the sensor. However, in some cases, such positions may not be available in the above depth range, then the seeds should be planted in depths which exhibit the highest RSM value.

### 2.5. Validation of the Variable Depth Planting Method for Maize Planting

**Figure 5:**
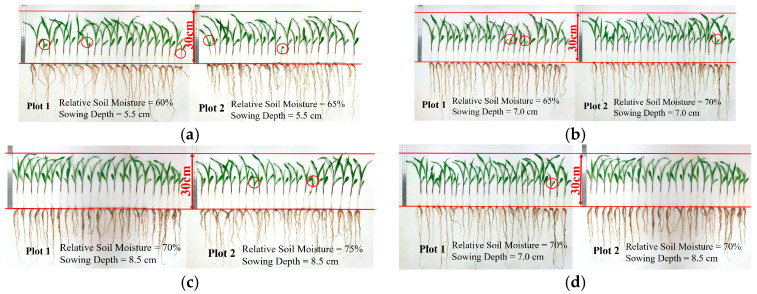
Maize growth under different validation treatments: (**a**) TCP-a; (**b**) TCP-b; (**c**) TCP-c; and (**d**) VDP-a.

Maize growth at seedling stage under different validation treatments is shown in Figure 5. Seedlings shorter than 20 cm were marked by red circles in the figures. It is obvious that the uniformity of height and the vertical growth of seedlings are better in the variable depth planting (VDP) treatment. Moreover, the calculation results of seedling growth indexes for the validation treatments are shown in Table 6. When seeds were planted at different depths in the range of 5.5 to 8.5 cm in the VDP method, the adequate moisture environment reduced the adverse effects of variable sowing depth on the emergence of maize seedlings. Maize seeds had enough energy to carry out seedling emergence activities; hence, the higher result of emergence rate (*ER*) was obtained under the VDP method. Furthermore, although seeds sown by the traditional constant-depth planting (TCP) method had the same distance to break out of the ground, differences in soil moisture at seeding sites caused the maize seeds to enter the emergence stage asynchronously. It could be the reason why the calculated results of uniformity (*U_n_*) for this planting method were significantly lower than those of the VDP method. Overall, *ER*, mean leaf number (*MLN*), mean plant height (*MPH*), and *U_n_* increased by 9.31%, 17.92%, 3.72%, and 25.29% under the VDP method, indicating that it is a more reasonable sowing pattern with ability to improve maize seedling growth in the dryland with uneven water distribution.

## 3. Discussion

### 3.1. Functional Interplay Between Soil Moisture and Sowing Depth

The quadratic models revealed a significant interaction between relative soil moisture (RSM) and sowing depth (SD), indicating that maize seedling performance is governed by a synergistic interplay rather than by purely additive effects. Consistent with prior studies conducted in rain-fed regions, RSM exerted a dominant influence on emergence rate (ER), mean plant height (MPH), and uniformity (Uₙ), underscoring the role of soil water as the primary limiting factor [22,23,24]. In parallel, SD strongly affected ER and mean leaf number (MLN), suggesting that mechanical resistance—both depth-dependent factors—must be adequately moderated to promote plumule development [25,26]. The observed optimal window (RSM near 70% and SD between 5.5 and 8.5 cm) promotes seedling emergence by achieving a critical balance. Adequate moisture at these depths ensures sufficient water availability for seed imbibition and enzymatic activation, while minimizing the mechanical resistance that the plumule must overcome to reach the soil surface. Shallower sowing (<5.5 cm) risks moisture stress due to higher evaporation, whereas deeper sowing (>8.5 cm) increases energy expenditure for mesocotyl elongation, potentially depleting seed reserves before emergence, especially under marginal moisture conditions. This explains why the positive effect of increasing soil moisture is substantially reduced outside this depth window.

### 3.2. The Variable Depth Planting Method in Precision Agriculture

The variable depth planting (VDP) method operationalizes this understanding by using maps of soil water variability to place seeds at the depth where relative moisture is closest to 70% within the 5.5–8.5 cm optimal window. By guaranteeing adequate water during imbibition and germination, VDP accelerates and synchronizes seedling emergence. Compared with traditional constant-depth planting (TCP), VDP increased ER, MLN, MPH, and Uₙ by 9.31%, 17.92%, 3.72%, and 25.29%, respectively, demonstrating a more rational sowing strategy for dryland maize under patchy water availability. These early-growth traits are strongly correlated with final grain yield [27,28,29]. However, environmental variability must be considered. Soil temperature, which co-varies with depth, can affect germination speed, and its interaction with moisture in the VDP context warrants further study. Furthermore, while heavy post-sowing rainfall could temporarily negate the VDP advantage by altering seed-zone moisture, this risk can be managed by integrating short-term weather forecasts into the planting decision. Implementing VDP on-farm will require affordable on-the-go soil moisture sensors and real-time depth-actuation systems—technologies that are under intensive development and expected to reach commercial maturity within a few years.

### 3.3. Limitations and Future Research

(1) Generalizability Across Soils and Sites: The experiment was conducted on a single sandy-loam soil. The optimal RSM–SD window may shift in soils with higher clay or silt contents due to differences in water retention, bulk density, and impedance, or in soils affected by salinity. Therefore, the conclusions are currently texture-specific. Multi-site experiments across contrasting soil orders and climates are essential to calibrate and develop robust, adaptable VDP algorithms.

(2) Beyond the Seedling Stage: The experimental period was limited to 10 days post-sowing, capturing key seedling establishment traits. While these are predictive of final performance, the long-term agronomic impact of VDP on root architecture, water use efficiency(WUE), and final grain yield remains to be quantified. Future studies extend the measurement period to encompass the full growing season to validate the yield benefits and resource use efficiencies implied by the strong early advantages.

(3) System Integration and Environmental Dynamics: As noted, VDP performance relies on planting immediately after soil moisture mapping to avoid evaporation losses. Future prototypes should integrate real-time sensing and actuation within a single pass. Furthermore, a comprehensive analysis is needed to evaluate how variable environmental factors like diurnal temperature fluctuations and unpredictable rainfall events affect the robustness of the VDP strategy across seasons.

(4) Economic and Environmental Analysis: While VDP promises to boost stand uniformity and potentially reduce the need for re-sowing or supplemental irrigation, a full life-cycle assessment is required to confirm its economic viability and contributions to carbon–water savings under varying market and agro-ecological conditions.

## 4. Materials and Methods

### 4.1. Experiment for Constructing the Framework of Variable Depth Planting Method

#### 4.1.1. Experiment Site and Soil Characteristics

The experiment was conducted at the China Agricultural University in Beijing (116°21′19′′ E, 40°0′11′′ N) during a maize growing season under temperate monsoon climate conditions. The average temperature during the experiment was 25 °C. The proportion of sand (63–2000 μm), silt (2–63 μm), and clay (<2 μm) in the soil were 67.55%, 30.48%, and 1.97%, respectively. The soil texture was classified as sandy-loam based on the World Reference Base for Soil Resources (WRB) 2022 [30] and belonged to the main soil texture in dryland maize producing areas of northern China [31]. The soil volumetric mass of the experiment site was 1.3 ${g/cm}^{3}$, and the water holding capacity was measured to be 19%.

#### 4.1.2. Experiment Design

Two factors were involved in the experiment: relative soil moisture (RSM) and sowing depth (SD). The levels of RSM in the experiment were set at 60%, 65%, 70%, and 75%, including the soil moisture conditions common to dryland maize cultivation. Considering the agronomic requirements of maize planting, the levels of SD were set at 2.5 cm, 4.0 cm, 5.5 cm, 7.0 cm, and 8.5 cm, respectively. Then, the 4 × 5 factorial experiment was arranged in a randomized complete block design with three replications. Test factors and levels are shown in Table 7.

The maize cultivar ‘Zhengdan 958’ (ZD958, Henan Academy of Agricultural Sciences, China), which is planted most commonly in the area, was used as experimental material. The amount of water applied to each block was based on the difference between the current RSM and the RSM to be achieved. Then, the soil was evenly mixed so that the water distributed in it could become more uniform. There were 20 treatments in total in the experiment. In each treatment, 24 maize seeds were seeded manually at the position of the set SD level in four rows with a row spacing of 15 cm. The soil was compacted evenly, with the aim of providing uniform and well-contact for the seeds with it. Maize development always depends on the nutrients stored inside the seed before the BBCH 13 stage; therefore, the main external environmental factors affecting maize growth before this period are soil moisture and sowing depth. To obtain a better experimental result, the experiment was ended by the 10th day, when more than 50% of the maize seedlings had grown to the BBCH 13 stage (the third leaf on the main stalk sticking out about 2 cm).

#### 4.1.3. Experiment Site and Soil Characteristics

##### Soil Properties

Soil texture was measured by MS3000 Laser Particle Sizer (Malvin Instrument Co., Ltd., the Netherlands) through the method proposed by Sedláčková et al. [32]. Before sowing, 100 cm^3^ soil cores were used to obtain evenly mixed soil samples, these samples were used to determine the current gravimetric soil moisture (GSM) by 105 °C oven-drying for 24 h, as well as the soil water holding capacity (WHC) following the description of Muratore et al. [33]. At the end of the experiment, soil samples were collected at depths of 2.5, 4.0, 5.5, 7.0, and 8.5 cm to measure the GSM by the same method. In this study, obtained values of GSM were transferred to RMS through Equation (1):

(1) $$RSM/\%=\frac{GSM}{WHC}\times 100$$

##### Evaluation Indexes of Maize Growth

After sowing, the number of seeds that emerged each day (seedling height >2 cm) was counted at the same time. When the experiment ended, the plant height was measured as the extended leaf heights (i.e., distance from the soil surface to the uppermost extended leaf tip) with a ruler, accurate to 0.01 cm. In addition, the number of leaves on all plants was counted. In this experiment, emergence rate (*ER*), mean leaf number (*MLN*), mean plant height (*MPH*), and uniformity (*U_n_*) were used as indexes to evaluate maize growth traits. They were calculated based on the relevant equations from [34,35], restated here as Equations (2)–(5):

(2) $$ER=\frac{n}{m}\times 100$$

(3) $$MLN=s=\frac{\Sigma s}{m}$$

(4) $$MPH=h=\frac{\Sigma h}{n}$$

(5) $$U_{n}=\frac{h}{S}=\frac{h}{\sqrt{\left((\Sigma h^{2}-{\left(\Sigma h\right)}^{2}/n)/((n-1))\right)}}$$

where the n is the total number of seeds emerged, m is the total number of seeds sown, s is the number of leaves for a single plant, h is the height of a single plant (cm), and S is the standard deviation of plant height (cm).

#### 4.1.4. Data Analysis

Calculation results of *ER*, *MLN*, *MPH,* and *U_n_* among *RSM* and *SD* levels were subjected to significance analysis and response surface analysis. Comparisons of means among different SD levels were performed with the level of *p* ≤ 0.05 using the Duncan test. After that, within the obtained suitable SD range for maize growth, comparisons of means among different RSM levels were also performed through the Duncan test to acquire the optimal RSM value. The Design-Expert 8.0.6 (Stat-Ease, Minneapolis, MN, USA) analytical software was used for all of the statistical analyses.

### 4.2. Validation Experiment

#### Experiment Method

After constructing the variable depth planting (VDP) method for dryland maize cultivation, a validation experiment was designed to evaluate the performance of the proposed method in improving maize seedling traits for two adjacent fields with uneven water distribution. Relative soil moisture (RSM) data were obtained at depths of 5.5, 7.0, and 8.5 cm in the two fields, and the results are shown in Table 8. Then, the main treatments were as follows: (1) placing seeds in locations where sowing depth (SD) was the same while RSM was variable, as well as (2) placing seeds in locations where RSM was optimal but SD was diverse.

Based on the above RSM data and the designed treatments, the specific combinations of SD and RSM for each plot are listed in Table 9.

The results of the overall *ER*, *MLN*, *MPH,* and *U_n_* calculated for the two plots were used as evaluation standards to select the more effective maize planting method in terms of improving seedling growth traits. The experiment site, material, and measurement methods were the same as described above.

## 5. Conclusions

This study developed a site-specific variable depth planting (VDP) method to optimize maize seedling establishment in the drylands of northern China. The main conclusions are as follows:

(1) A significant interaction between soil moisture and sowing depth was identified, with optimal seedling performance occurring within a synergistic depth–moisture window (SD: 5.5–8.5 cm; target RSM: 70%).

(2) The VDP method, which places seeds at depths where soil moisture is closest to 70% within this range, significantly improved early-growth traits—emergence rate, leaf number, plant height, and uniformity increased by 9.31–25.29% compared to conventional planting.

(3) This approach effectively utilizes spatial soil moisture variability to enhance seedling establishment and is compatible with precision planters equipped with real-time sensing and depth-control systems.

(4) Further research is needed to validate the method across diverse soil and climatic conditions, assess its full-season agronomic and economic impacts, and support broader on-farm adoption.

## Figures and Tables

**Table 1:** RSM variation at different SD levels. Abbreviations: SD, sowing depth; RSM, relative soil moisture.

SD/cm	RSM at the End of the Experiment/%			
	Initial RSM of 60%	Initial RSM of 65%	Initial RSM of 70%	Initial RSM of 75%
2.5	20	21	22	24
4.0	28	29	30	32
5.5	34	38	41	43
7.0	42	44	46	48
8.5	43	46	48	51

**Table 2:** Regression equations for each maize seedling growth index. Abbreviations: ER/*Y*_1_, emergence rate; MLN/*Y*_2_, mean leaf number; MPH/*Y*_3_, mean plant height; $U_{n}$/*Y*_4_, uniformity; *X*_1_, relative soil moisture; *X*_2_, sowing depth.

Maize Seedling Growth Indexes	Regression Equations
ER	Y_1_ = −175.65600 + 6.77358 X_1_ + 12.62832 X_2_ − 0.13780 X_1_X_2_ − 0.04328 X_1_^2^ − 0.25897 X_2_^2^
MLN	Y_2_ = −10.80738 + 0.33230 X_1_ + 0.72877 X_2_ − 5.88000 × 10^−3^ X_1_X_2_ − 2.20000 ×10^−3^ X_1_^2^ − 0.02373 X_2_^2^
MPH	Y_3_ = −121.58531 + 3.90670 X_1_ +1.52899 X_2_ − 0.01536 X_1_X_2_ − 0.02638 X_1_^2^ − 0.07238 X_2_^2^
U_n_	Y_4_ = −338.24438 + 9.73678 X_1_ +4.59507 X_2_ − 0.03836 X_1_X_2_ − 0.06660 X_1_^2^ − 0.11754 X_2_^2^

**Table 3:** Significance analysis for each maize seedling growth index. Abbreviations: ER, emergence rate; MLN, mean leaf number; MPH, mean plant height; $U_{n}$, uniformity; *X*_1_, relative soil moisture; *X*_2_, sowing depth.

Sources	ER		MLN		MPH		U_n_	
	F-Value	*p*-Value	F-Value	*p*-Value	F-Value	*p*-Value	F-Value	*p*-Value
Model	23.07	<0.0001 **	20.05	<0.0001 **	9.28	0.0005 **	8.48	0.0007 **
*X*_1_	15.95	0.0013 **	0.71	0.4140	32	<0.0001 **	26.31	0.0002 **
*X*_2_	17.57	0.0009 **	58.45	<0.0001 **	6.26	0.0254 *	6.73	0.0212 *
*X*_1_ *X*_2_	45.61	<0.0001 **	12.59	0.0032 **	0.50	0.4915	0.61	0.4481
*X*_1_^2^	20.00	0.0005 **	7.83	0.0142 *	6.54	0.0228 *	8.16	0.0127 *
*X*_2_^2^	16.24	0.0012 **	20.66	0.0005 **	1.12	0.3085	0.58	0.4603

Note: **: *p* ≤ 0.01, highly significant; *: *p* ≤ 0.05, significant; *p* ≥ 0.05, not significant.

**Table 4:** Comparison results of maize seedling growth indexes under different sowing depth (SD) levels. Abbreviations: ER, emergence rate; MLN, mean leaf number; MPH, mean plant height; $U_{n}$, uniformity. Columns sharing the same letter are not significantly different (*p* ≥ 0.05).

SD/cm	ER/%	MLN/pc	MPH/cm	Un
2.5	90.0833 b	2.2433 c	22.0725 a	18.2833 b
4.0	91.2842 ab	2.3892 b	21.2133 ab	17.9608 b
5.5	93.9317 a	2.6933 a	22.5308 a	22.6108 a
7.0	93.8967 a	2.6983 a	20.4733 b	21.1567 a
8.5	92.3617 ab	2.6200 a	20.1633 b	22.0325 a

**Table 5:** Comparison results of maize seedling growth indexes under different relative soil moisture (RSM) levels. Abbreviations: ER, emergence rate; MLN, mean leaf number; MPH, mean plant height; $U_{n}$, uniformity. Columns sharing the same letter are not significantly different (*p* ≥ 0.05).

RSM/%	ER/%	MLN/pc	MPH/cm	Un
60	92.5911 a	2.6544 ab	18.7244 c	17.0889 c
65	93.9899 a	2.6878 ab	20.8511 b	20.6244 b
70	95.2133 a	2.7922 a	22.3578 a	25.8511 a
75	91.7933 a	2.5478 b	22.2900 a	24.1689 a

**Table 6:** Results of different validation treatments. Abbreviations: TCP, traditional constant-depth planting; VDP, variable depth planting; ER, emergence rate; MLN, mean leaf number; MPH, mean plant height; $U_{n}$, uniformity.

Planting Method	Serial No.	Growth Indexes			
		ER/%	MLN/pc	MPH/cm	U_n_
TCP	a	89.58	2.40	20.96	18.82
	b	95.83	2.75	21.39	20.37
	c	93.75	2.71	21.35	21.46
VDP	a	97.92	2.83	21.74	23.58

**Table 7:** Test factor level. Abbreviations: SD, sowing depth; RSM, relative soil moisture; GSM, gravimetric soil moisture; VSM, volumetric soil moisture.

Level	Factor		GSM/% (RSM to GSM)	VSM/% (RSM to GSM)
	SD/cm	RSM/%		
1	2.5	60	11.4	14.82
2	4.0	65	12.35	16.06
3	5.5	70	13.3	17.29
4	7.0	75	14.25	18.53
5	8.5	\	\	\

**Table 8:** RSM data at depths of 5.5, 7.0, and 8.5 cm in two fields. Abbreviations: SD, sowing depth; RSM, relative soil moisture.

SD/cm	Field 1	Field 2
	RSM/%	
5.5	60	65
7.0	65	70
8.5	70	75

**Table 9:** Main treatments in the validation experiment. Abbreviations: TCP, traditional constant-depth planting; VDP, variable depth planting; SD, sowing depth; RSM, relative soil moisture.

Planting Method	Serial No.	Plot 1		Plot 2	
		SD/cm	RSM/%	SD/cm	RSM/%
TCP	a	5.5	60	5.5	65
	b	7.0	65	7.0	70
	c	8.5	70	8.5	75
VDP	a	7.0	70	8.5	70

## Data Availability

Data are contained within the article.

## Abbreviations

The following abbreviations are used in this manuscript:

RSM	Relative soil moisture
GSM	Gravimetric soil moisture
WHC	Water holding capacity
WUE	Water use efficiency
SD	Sowing depth
TCP	Traditional constant-depth
VDP	Variable depth planting
ER	Emergence rate
MLN	Mean leaf number
MPH	Mean plant height
U_n_	Uniformity
